# Cytotoxicity of Triorganophosphinegold(I) n-Mercaptobenzoates, n = 2, 3 and 4

**DOI:** 10.1155/MBD.2002.303

**Published:** 2002

**Authors:** Dick de Vos, Douglas R. Smyth, Edward R. T. Tiekink

**Affiliations:** 1 Medical Department Pharmachemie B.V. Haarlem NL-2003 The Netherlands; 2 Department of Chemistry The Univeristy of Adelaide 5005 Australia; 3 Department of Chemistry National University of Singapore Singapore 117543 Singapore

## Abstract

The results of cytotoxicity trials against a panel of seven human cell lines for a series of triorganophosphinegold(I) 3- and 4-mercaptobenzoates are reported. While the new compounds show moderate to high toxicity, their potencies are inferior to those reported previously for their isomeric 2- mercaptobenzoate derivatives. The results therefore suggest a structure-activity relationship in that the 2-isomeric species are more active, particularly against the non-small cell lung cancer and renal cancer cell lines, results that may indicate some selectivity in their cytotoxic profile.


### CYTOTOXICITY OF TRIORGANOPHOSPHINEGOLD(I) n-MERCAPTOBENZOATES, n = 2, 3 AND 4

Dick de Vos,[1] Douglas R. Smyth[2] and Edward R.T. Tiekink*,[3]

[1] Medical Department, Pharmachemie B.V., NL-2003 Haarlem, The Netherlands
[2] Department of Chemistry, The Univeristy of Adelaide, Australia 5005
[3] Department of Chemistry, National University of Singapore, Singapore 117543
<chmtert@nus.edu.sg>

**Abstract**
The results of cytotoxicity trials against a panel of seven human cell lines for a series of triorganophosphinegold(I) 3- and 4-mercaptobenzoates are reported. While the new compounds show moderate to high toxicity, their potencies are inferior to those reported previously for their isomeric 2-mercaptobenzoate derivatives. The results therefore suggest a structure-activity relationship in that the 2-isomeric species are more active, particularly against the non-small cell lung cancer and renal cancer cell lines, results that may indicate some selectivity in their cytotoxic profile.

**Introduction**
Gold complexes are well known to possess significant anti-arthritic activity and several gold thiolates are currently used clinically in the treatment of that disease [1-3]. In addition to this biological profile, several gold(I), as well as gold(III) species, have proven to display cytotoxicity and anti-tumour activity as summarised in a recent survey [4]. In this context, one of the active classes of compounds features a linear P-Au-S entity as found in the anti-arthritic drug, auranofin (triethylphosphinegold(I) tetraacetatothioglucose). Our work in this area has focussed on making derivatives of auranofin, some of which display significant cytotoxicity and anti-tumour activity [5-8]. A difficulty encountered with these compounds has been their limited aqueous solubility. In order to overcome this limitation, thiols containing water-solubilising groups have been coordinated to the phosphinegold(I) entity. Amongst the thiols chosen for investigation was 2-mercaptobenzoic acid. These derivatives displayed moderate to high cytotoxicity profiles compared to standards such as cisplatin, doxorubicin, methotrexate, *etc.* [9]. In particular, significant cytotoxicity was found against the non-small cell lung cancer and renal cancer cell lines. As a continuation of work in this area, some 3- and 4-mercaptobenzoates have been prepared and subjected to screening for their cytotoxicity profiles. The results of this study are reported herein.

**Results and Discussion**
The triorganophosphinegold(I) n-mercaptobenzoates, $R_3PAu$(n-mbaH), have been synthesised using established procedures involving the metathesis of the triorganophosphinegold(I) chloride precursor with the appropriate n-mercaptobenzoic acid in the presence of base. Characterisation data are collected in the Experimental section. A number of the n-mercaptobenzoates compounds have been characterised crystallographically, by us [9 - 11] (*i.e.* n = 2 for R = Ph and Cy; n = 3 for R = Cy and Ph) and others [12] (*i.e.* n = 4 for R = Et).

The crystal structure of $Cy_3PAu$(4-mbaH) has been determined in the present study. A centrosymmetrically related pair of molecules is illustrated in Figure 1; selected geometric parameters are collected in the Figure caption. The gold atom exists in the expected linear geometry defined by the sulfur and phosphorus atoms so that the angle subtended at the gold atom is 175.06(4)°. The 4-mbaH ligand is essentially planar as seen in the magnitude of the O1/C1'/C1/C2 and O2/C1'/C1/C2 torsion angles of -174.3(4) and 5.0(7)°, respectively. Molecules associated via the familiar carboxylic acid dimer motif with the key parameters defining this association listed in the caption to Figure 1. The structure is in essential agreement with the $Et_3PAu$(4-mbaH) and o-$tol_3PAu$(4-mbaH) structures reported recently by Schmidbaur *et al.* [12].

A previous report [9] summarising the cytotoxicity of a series of triorganophosphine 2-mercaptobenzoates showed high levels of activity, in particular against the non-small cell lung cancer (H226) and renal cancer (A498) cell lines. These results are reproduced in Table 1. Subsequently, biological trials for some 3- and 4-mercaptobenzoate derivatives of $R_3PAu$, for R = Et, Cy & Ph, were also conducted.

As seen from Table 1, a general structure-activity relationship can be established in that the 2-mercaptobenzoate compounds are generally more active than their 3- and 4- isomeric counterparts. The most cytotoxic compound in the series investigated was the $Et_3PAu$(2-mbaH) compound which displayed a $ID_{50}$ value of 43 ng/ml against the IGROV cell line but, doxorubicin and methotrexate were more active. High activity against the H226 and A498 cell lines was only observed for the 2-mbaH series perhaps indicating some selectivity in activity. It would be of some interest to ascertain whether this activity is maintained *in vivo* leading to anti-tumour activity.

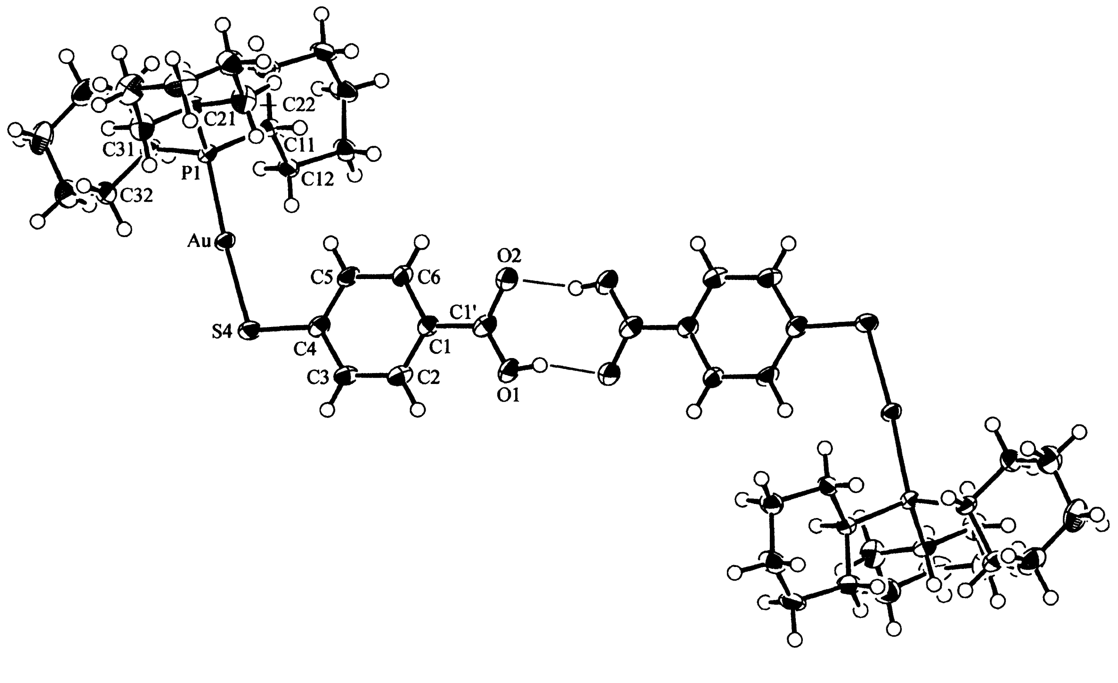

**Figure 1.** The centrosymmetric hydrogen-bonded dimer of $Cy_3PAu(4\text{-}mbaH)$ (50% probability level). Selected bond distances and angles: Au-S4 2.308(2), Au-P1 2.273(1), S4-C4 1.759(5), C1'-O1 1.280(5), C1'-O2 1.250(5) Å;, S4-Au-P1 175.06(4), Au-S4-C4 105.53(15)°. Parameters associated with the hydrogen bonding interaction are O1-H...O2$^{i}$ 1.81 Å, O1...O2$^{i}$ 2.628(5) Å and an angle subtended at H of 166°; symmetry operation -1-*x*, 1-*y*, 1-*z*.

**Table 1.** *In vitro* $ID_{50}$ values (ng/ml) for $R_3PAu(n\text{-}mbaH)$, n = 2, 3 & 4 and R = Et, Cy & Ph, using the microculture sulforhodamine B (SRB) test as cell viability, and for the reference compounds: doxorubicin (DOX), cisplatin (CPT), 5-fluorouracil (5-FU), methotrexate (MTX) and etoposide (ETO)

| R | n | Cell Line | | | | | | |
|---|---|---|---|---|---|---|---|---|
| | | MCF7 | EVSA-T | WIDR | IGROV | M19MEL | A498 | H226 |
| Et | 2 [9] | 405 | 110 | 984 | 43 | 118 | 65 | 69 |
| Cy | 2 [9] | 411 | 225 | 772 | 182 | 292 | 95 | 97 |
| Ph | 2 [9] | 791 | 356 | 982 | 117 | 298 | 151 | 173 |
| Cy | 3 | 806 | 1046 | 949 | 283 | 403 | 697 | 848 |
| Ph | 3 | 1065 | 1287 | 957 | 280 | 797 | 1274 | 960 |
| Et | 4 | 1145 | 1197 | 1238 | 67 | 375 | 484 | 864 |
| Cy | 4 | 973 | 1353 | 976 | 338 | 592 | 743 | 923 |
| Ph | 4 | 858 | 940 | 553 | 148 | 322 | 348 | 586 |
| standards | | | | | | | | |
| DOX | | 8 | 11 | 10 | 16 | 60 | 90 | 199 |
| CPT | | 422 | 967 | 699 | 558 | 169 | 2253 | 3269 |
| 5-FU | | 475 | 225 | 750 | 442 | 297 | 143 | 340 |
| MTX | | 5 | <3 | 18 | 23 | 7 | 37 | 2287 |
| ETO | | 317 | 150 | 2594 | 505 | 580 | 1314 | 3934 |

**Experimental**

*Preparations*

The $R_3PAu$(n-mbaH) compounds were prepared by literature procedures established for the triorganophosphine 2-mercaptobenzoates [9]. Characterisation details for the n = 3, R = Cy and Ph compounds are available in the literature [10]. During the course of the present study, the preparation and characterisation of the n = 4, R = Et and Ph derivatives were reported [12]. Full details for the n = 4, R = Cy compound are presented below as well as previously unreported spectroscopic details for the n = 4, R = Et and Ph compounds.

$Cy_3PAu$(4-mbaH): Yield 71% of colourless crystals that decomposed above 250 °C. Crystals for the X-ray study were grown by the slow evaporation of a $CH_2Cl_2$/EtOH (1/1) solution of the compound. C, 47.5; H, 6.1. $C_{25}H_{38}AuO_2PS$ requires C, 47.6; H, 6.1%. IR (KBr disk): ν(C=O) 1674(s) and ν(O-H) 2500 - 3200 (br) $cm^{-1}$. $^1H$ NMR ($d_6$-DMSO): δ 7.58 (d, 8.4 Hz, H2 of 4-mbaH); 7.42 (d, 8.4 Hz, H3 of 4-mbaH); 12.5 (br, $CO_2H$). $^{13}C\{^1H\}$ NMR ($d_6$-DMSO): δ 124.9, 128.8, 131.0, 151.8, 167.2 (4-mbaH: C1, C2, C3, C4, $CO_2$); 32.4 (d, 28.3 Hz, Cα); 26.3 (d, 12.1 Hz, Cβ); 30.3 (Cγ), 25.5 ppm (Cδ). $^{31}P$ NMR ($d_6$-DMSO): δ 59.2 ppm.

$Et_3PAu$(4-mbaH): IR (KBr disk): ν(C=O) 1688(s) and ν(O-H) 2500 - 3200 (br) $cm^{-1}$. $^{13}C\{^1H\}$ NMR ($d_6$-DMSO): δ 124.4, 128.8, 131.1, 151.9, 167.2 (4-mbaH: C1, C2, C3, C4, $CO_2$); 17.0 (d, 33.5 Hz, Cα); 9.0 (Cβ). $^{31}P$ NMR ($d_6$-DMSO): δ 39.9 ppm.

$Ph_3PAu$(4-mbaH): IR (KBr disk): ν(C=O) 1669 (s) and ν(O-H) 2500 - 3200 (br) $cm^{-1}$. $^{13}C\{^1H\}$ NMR ($d_6$-DMSO): δ 125.1, 129.1, 131.2, 150.8, 167.2 (4-mbaH: C1, C2, C3, C4, $CO_2$); 128.7 (d, 57.0 Hz, Cα); 133.7 (d, 13.7 Hz, Cβ); 129.6 (d, 11.5 Hz, Cγ), 132.0 ppm (d, 2.3 Hz, Cδ). $^{31}P$ NMR ($d_6$-DMSO): δ 38.7 ppm.

*Biological screening*

The following human tumour cell lines were used: MCF7 (breast cancer), EVSA-T (breast cancer), WIDR (colon cancer), IGROV (ovarian cancer), M19MEL (melanoma), A498 (renal cancer), and H226 (non-small cell lung cancer). Cell lines WIDR, M19 MEL, A498, IGROV and H226 belong to the currently used anti-cancer screening panel of the National Cancer Institute, USA [13]. The MCF7 cell line is estrogen receptor (ER)+/ progesterone receptor (PgR)+ and the cell line EVSA-T is (ER)-/(PgR)-.

Prior to the experiments, a mycoplasma test was carried out on all cell lines and found to be negative. All cell lines were maintained in a continuous logarithmic culture in RPMI 1640 medium with Hepes and phenol red. The medium was supplemented with 10% FCS, penicillin 100 IU/ml and streptomycin 100 μg/ml. The cells were mildly trypsinised for passage and for use in the experiments.

*Chemicals*

RPMI and FCS were obtained from Life Technologies (Paisley, Scotland). SRB, DMSO, penicillin and streptomycin were obtained from Sigma (St. Louis MO, USA), TCA and acetic acid from Baker BV (Deventer, NL) and PBS from NPBI BV (Emmer-Compascuum, NL).

*Experimental Procedures*

The test and reference compounds were dissolved to a concentration of 250000 ng/ml in full medium, by 20 fold dilution of a stock solution which contained 1 mg compound/200μg after having been dissolved first in in DMSO solution. Cytotoxicity was estimated by the microculture sulforhodamine B (SRB) test [14].

**Table 2.** Crystal data for $Cy_3PAu$(4-mbaH)

| | | | |
|---|---|---|---|
| Empirical formula | $C_{25}H_{38}AuO_2PS$ | Formula weight | 630.57 |
| Diffractometer | Rigaku AFC7R | Space group | monoclinic, $P2_1/c$ |
| Unit cell dimensions | $a$ 12.257(6) Å | Volume, $Å^3$ | 2532(2) |
| | $b$ 16.559(9) Å $\beta$ 98.69(3) ° | $Z$ | 4 |
| | $c$ 12.621(4) Å | $D_{calc}$, g $cm^{-3}$ | 1.654 |
| μ (Mo-$K\alpha$), $cm^{-1}$ | 59.94 | $F$(000) | 1256 |
| θ range, ° | 3.3 - 27.5 | Temperature, K | 173 |
| Reflections collected | 5805 | Reflections with $I>2\sigma(I)$ | 4115 |
| No. parameters refined | 272 | $R$ [$I>2\sigma(I)$] | 0.025 |
| $wR$ (all data) | 0.060 | Goodness-of-fit on $F^2$ | 1.01 |
| Weighting scheme | $w = [\sigma^2+(0.0287P)^2]^{-1}$ where $P = (F_o^2 + 2F_c^2)/3$ | | |
| Diff. hole and peak, $eÅ^{-3}$ | -0.76 to 1.41 | CCDC deposition no. | 181875 |
| Programs | teXsan [16], DIRDIF [17], SHELXL-97 [18], ORTEPII [19] | | |

The experiment was started on day 0. On day 0, 150 μl of trypsinised tumour cells (1500-2000 cells/well) were plated in 96-wells flatbottom microtiter plates (Falcon 3072, BD). The plates were preincubated for 48 h at 37 °C, 8.5% $CO_2$ to allow the cells to adhere. On day 2, a three-fold dilution sequence of ten steps was made in full medium, starting with the 250000 ng/ml stock solution. Every dilution was used in quadruplicate by adding 50 μg to a column of four wells. This results in a highest concentration of 62500 ng/ml present in column 12. Column 2 was used for the blank. To column 1 PBS was added to diminish interfering evaporation. On day 7, the incubation was terminated by washing the plate twice with PBS. Subsequently the cells were fixed with 10% trichloroacetic acid in PBS and placed at

4 °C for one hour. After five washings with tap water, the cells were stained for at least 15 minutes with 0.4% SRB dissolved in 1% acetic acid. After staining the cells were washed with 1% acetic acid to remove the unbound stain. The plates were air-dried and the bound stain was dissolved in 150 µl 10 mM Tris-base. The absorbance was read at 540 nm using an automated microplate reader (Labsystems Multiskan MS).
Data were used for construction of concentration-response curves and determination of the $ID_{50}$ value by use of Deltasoft 3 software.

*Crystallography*

A colourless crystal with dimensions 0.16 x 0.16 x 0.38 mm was used in the X-ray diffraction study. An empirical absorption correction was applied [15]. The maximum residual electron density peak was located in the vicinity of the gold atom.

**Acknowledgments**

The Australian Research Council is thanked for support. D.R.S. was the grateful recipient of an University of Adelaide Postgraduate Research Award. The National University of Singapore is thanked for on-going support of this research (143-000-139-112).

**References**

[1] R.V. Parish, *Interdiscip. Sci. Rev.* 1992, **17**, 221.
[2] Shaw, C.F. III, *Chem. Rev.* **99**, 1999, 2589.
[3] E.R.T. Tiekink and M.W. Whitehouse, In Handbook of Metal-Ligand Interactions in Biological Fluids. G. Berthon (Ed.), Marcel Dekker Inc., 1995, Vol. 2, 1266.
[4] E.R.T. Tiekink, *Crit. Rev. Oncol. Hemato.* 2002, in press.
[5] E.R.T. Tiekink, P.D. Cookson, B.M. Linahan and L.K. Webster, *Metal-Based Drugs*, 1994, **1**, 299.
[6] L.K. Webster, S. Rainone, E. Horn and E.R.T. Tiekink, *Metal-Based Drugs*, 1996, **3**, 63.
[7] E.R.T. Tiekink, In Ph. Collery, J. Corbella, J.L. Domingo, J.C. Etienne and J.M. Llobet (Eds) Metal Ions in Biology and Medicine. 1996, **4**, 693.
[8] D. Crump, G. Siasios and E.R.T. Tiekink, *Metal-Based Drugs*, 1999, **6**, 361.
[9] D. de Vos, P. Clements, S.M. Pyke, D.R. Smyth and E.R.T. Tiekink, *Metal-Based Drugs*, 1999, **6**, 31.
[10] D.R. Smyth, B.R. Vincent and E.R.T. Tiekink, *Z. Kristallogr.* 2001, **216**, 298.
[11] D.R. Smyth, B.R. Vincent and E.R.T. Tiekink, *Crystal Growth & Design*, 2001, **2**, 113.
[12] J.D.E.T. Wilton-Ely, A. Schier, N.W. Mitzel and H. Schmidbaur, *J. Chem. Soc., Dalton Trans*, 2001, 1058.
[13] M.R. Boyd, *Prin. Pract. Oncology*, 1989, **3**, 1.
[14] Y.P. Keepers, P.E. Pizao, G.J. Peters, J. van Ark-Otte, B. Winograd and H.M. Pinedo, *Eur. J. Cancer*, 1991, **27**, 897.
[15] N. Walker and D. Stuart, *Acta Crystallogr.* 1983, A**39**, 158.
[16] *teXsan*, *Single Crystal Structure Analysis Software*, Version 1.04 (1997), Molecular Structure Corporation, The Woodlands, TX, USA.
[17] P. T. Beurskens, G. Admiraal, G. Beurskens, W. P. Bosman, S. García-Granda, J. M. M. Smits and C. Smykalla, The DIRDIF program system, Technical Report of the Crystallography Laboratory, (1992), University of Nijmegen, The Netherlands.
[18] G. M. Sheldrick, SHELXL97, Program for the Refinement of Crystal Structures (1997), University of Göttingen, Germany.
[19] C. K. Johnson, ORTEP. Report ORNL-5138 (1976), Oak Ridge National Laboratory, TN, USA.

**Received: April 2, 2002 – Accepted: April 10, 2002 –
Accepted in publishable format: April 15, 2002**

